# Biochemical Analysis of Ferritin and D-dimer in COVID-19 Survivors and Non-survivors

**DOI:** 10.7759/cureus.45389

**Published:** 2023-09-17

**Authors:** Abdulrahman Hakami, Tahani Altubayqi, Entsar A Qadah, Basem Zogel, Samar M Alfaifi, Eman Refaei, Ahmed Sayed, Luai Alhazmi, Maram Sayegh, Abdullah Alamer, Areej S Areeshi, Duaa Hakami

**Affiliations:** 1 Department of Medicine, Faculty of Medicine, Jazan University, Jazan, SAU; 2 Department of Medicine, Jazan General Hospital, Jazan Health Affairs, Ministry of Health, Jazan, SAU

**Keywords:** d-dimer, serum ferritin, biochemical parameters, rt-pcr, sars-cov-2, covid-19

## Abstract

Background

COVID-19 is a major cause of illness and mortality. The management of COVID-19-related illnesses might change if variables connected to their severity and the requirement for ICU admission could be found. The severity of COVID-19 might be efficiently predicted with several laboratory measures, such as ferritin levels and D-dimer analysis.

Objectives

This study aimed to evaluate the association between serum D-dimer and ferritin levels and their effects on mortality in patients with COVID-19.

Methods

This retrospective observational study included all patients with positive real-time polymerase chain reaction (RT-PCR) results for COVID-19 who were hospitalized in the Ministry of Health South Al-Qunfudah General Hospital between March and September 30, 2020. Their laboratory parameters, serum D-dimer, and ferritin levels were evaluated. IBM SPSS Statistics for Windows, Version 26.0 (released 2019; IBM Corp., Armonk, New York, United States) was used to analyze the data.

Results

A total of 318 COVID-19 patients were analyzed; 56.9% (n=181) were male and 43.1% (n=137) were female. Of these, 78.6% (n=250) survived, including 58% of men and 42% of women. The mean D-dimer was 2.1 mcg/mL (SD=3.16) and the mean ferritin was 698.59 ng/mL (SD=603.11). Non-recovered patients were substantially older (66.16 years old) and had higher D-dimer (5.46) mcg/mL and ferritin levels (992.96) ng/mL. Intubation length and gender did not affect survival. Of the non-survivors, 95.6% (n=239) were admitted to the ICU, and 50% (n=34) required mechanical ventilation.

Conclusions

COVID-19 infection mortality dramatically increased with older age and increased mean ferritin and plasma D-dimer values, which were significantly higher in COVID-19 non-survivors than in survivors. Therefore, assessing and monitoring these laboratory markers in the early stages of the disease may have a significant impact on preventing disease progression and death.

## Introduction

Coronavirus disease 2019 (COVID-19) is a viral disease that mostly affects the respiratory system. It is very contagious, and it has rapidly spread worldwide through close human interactions or the expelled respiratory droplets (coughs and sneezes) of infected people [1,2]. In March 2020, the World Health Organization labeled the disease a pandemic due to its global spread.

Fever, cough, dyspnea, and chest infiltrations are the most common symptoms of COVID-19 [3,4]. Although most infections are not dangerous, 15-20% of COVID-19 patients could develop a condition that requires intensive care unit (ICU) admission, such as respiratory arrest, shock, or multiple organ failure [5,6].

Acute respiratory failure and macrophage activation syndrome are the most common severe clinical manifestations of COVID-19. Both are characterized by the overproduction of pro-inflammatory cytokines, which cause endothelial damage to several vital organs, especially the lungs. Recent studies have found that COVID-19 led to extensive alveolar epithelial destruction, capillary damage or bleeding, hyaline membrane formation, alveolar septal fibrous proliferation, and pulmonary consolidation [7,8]. Laboratory parameters and inflammatory markers, including ferritin, D-dimer, lymphocytes, C-reactive protein, interleukins, and lactate dehydrogenase (LDH), have been shown to predict the severity of COVID-19 [9-12].

Our region witnessed a surge of COVID-19 infections requiring hospital admission, with high mortality. Therefore, this study aimed to identify the predictors of clinical outcome. Early identification could be paramount to the optimal utilization of medical resources to manage high-risk patients.

COVID-19 requires urgent identification of clinical and laboratory predictors of progression toward severe and critical cases. The laboratory parameters will enable risk stratification, guide interventional studies to target patients at enhanced risk of developing severe disease, and optimize the allocation of limited human and technical resources in the future waves of the pandemic. Furthermore, the identification of laboratory predictors will aim to identify suspected severe cases and those at high risk of mortality, which will improve clinical situational awareness [12]. This study will help to gain a better understanding of the disease process and prognosis in COVID-19 patients, as well as the factors that determine the outcome.

## Materials and methods

Materials and methods

This retrospective, medical record-based observational study included all confirmed COVID-19 patients hospitalized at the Ministry of Health South Al-Qunfudah General Hospital, Saudi Arabia, with positive results from the SARS-CoV-2 real-time reverse transcriptase polymerase chain reaction (RT-PCR) test on upper respiratory tract specimens. After approval by the ethics committee, the records of all patients admitted from March to September 30, 2020, were reviewed. Individuals under the age of 18 and COVID-19 patients who were not hospitalized were excluded.

Data collection

All verified COVID-19 patients treated as inpatients at the Ministry of Health South Al-Qunfudah General Hospital from March to September 30, 2020, had their medical records collected by two postgraduate residents, who entered the data into a predesigned proforma. Two professors from the departments of community medicine and pulmonary medicine examined and double-checked the data to ensure its validity. The information that was retrieved comprised demographic details including age and sex, admission type, use of a mechanical ventilator, time spent intubated while receiving mechanical ventilation, discharge (recovery or death), and laboratory test results (ferritin and D-dimer). Patients were chosen based on their clinical traits and admission requirements.

Data analysis

Microsoft Excel was used for data entry, cleaning, and coding. IBM SPSS Statistics for Windows, Version 26.0 (released 2019; IBM Corp., Armonk, New York, United States) was used for data analysis. Descriptive statistics were used to describe the demographic and clinical features of the study population. The mean and standard deviation (SD) were used to characterize continuous variables, whereas frequency and percentage were employed to characterize categorical variables. Using chi-square and t-tests, differences between recovered and non-recovered patients were evaluated. The hazard ratio for mortality was estimated using Cox regression analysis in a survival analysis. The concentrations of D-dimer and ferritin were examined as continuous variables. Age was evaluated as a categorical variable, with age groupings of 0 to 20, 21 to 40, 41 to 60, 61 to 80, and older than 80 years of age. Using Kaplan-Meier survival curves, the connections between age, D-dimer, ferritin concentrations, and mortality risk were illustrated. A p-value of less than 0.05 was considered statistically significant.

## Results

We included the data of 318 COVID-19 patients with a mean age of 53.55 years (standard deviation (SD)=17.88 years). Moreover, 56.9% (n=181) of the patients were males, and only 16.6% (n=48) of them were admitted to the ICU, and 14.2% %(n=45) needed the use of a mechanical ventilator. The mean duration under intubation with mechanical ventilation was 8.28 days (SD=5.3 days). Of all patients, 78.6% (n=250) survived (Table 1). According to the files of the patients, the mean D-dimer was 2.1 mcg/mL ranging between 0.18 and 13.43 (mcg/mL), with an SD of 3.16. The mean concentration of ferritin was 698.59 ng/mL, ranging between 5.97 and 2567.0 ng/mL, with an SD of 603.11 (Table 2).

**Table 1:** Demographic factors of participants (n=318)

		Count	%
Age		53.55 (SD=17.88)	
Sex	Male	181	56.9%
	Female	137	43.1%
Type of admission	Ward	241	83.4%
	ICU	48	16.6%
Use of mechanical ventilator	Yes	45	14.2%
	No	273	85.8%
Duration under intubation with mechanical ventilation (days)		8.28 (SD=5.30)	
Discharge (recovery or death)	Non-recovery	68	21.4%
	Recovery	250	78.6%

**Table 2:** Ferritin and D-dimer concentrations among patients

	Mean	Standard Deviation	Minimum	Maximum
D-dimer	2.10	3.16	0.18	13.43
Ferritin	698.59	603.11	5.97	2567.00

Non-surviving patients had a significantly higher age (66.16 years) compared with surviving patients (50.09 years; P=0.000). No significant difference existed between genders in recovery from COVID-19 (P=0.455). Of the non-surviving patients, 95.6% (n=239) were admitted to the ICU (P=0.000), and 50% (n=34) needed mechanical ventilation (P=0.000). However, the duration of intubation did not affect the mortality or survival rate (P=0.643). Non-surviving patients had significantly higher levels of D-dimer (5.46) mcg/mL and ferritin (992.96) ng/mL compared with surviving patients (1.55 and 578.3; P=0.000 and 0.000, respectively; Table 3).

**Table 3:** Differences between recovered and non-recovered patients

		Discharge (recovery or death)				
		Non-recovery		Recovery		P-value
		Count	%	Count	%	
Age	Mean (SD)	66.16	13.23	50.09	17.45	0.000
Sex	Male	36	52.9%	145	58.0%	0.455
	Female	32	47.1%	105	42.0%	
Type of admission	Ward	2	4.4%	239	98.0%	0.000
	ICU	43	95.6%	5	2.0%	
Use of mechanical ventilator	Yes	34	50.0%	11	4.4%	0.000
	No	34	50.0%	239	95.6%	
Duration under intubation with mechanical ventilation (days)	Mean (SD)	8.44	5.13	7.80	5.87	0.643
D-dimer	Mean (SD)	5.46	4.77	1.55	2.41	0.000
Ferritin	Mean (SD)	992.96	607.78	619.81	578.30	0.000

**Figure 1:**
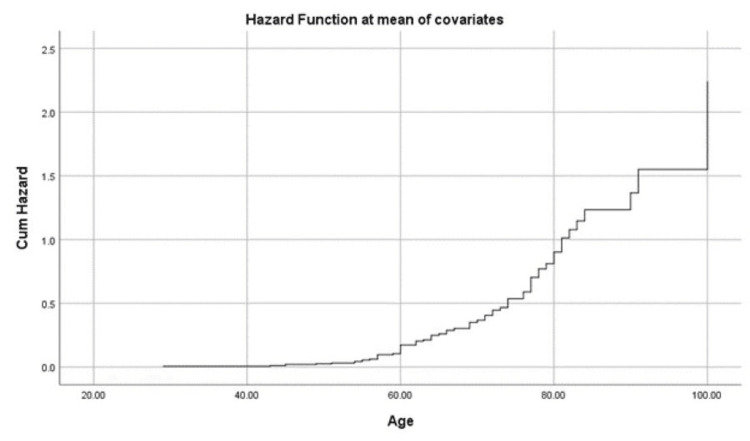
Mortality hazard among patients in association with D-dimer concentration

**Figure 2:**
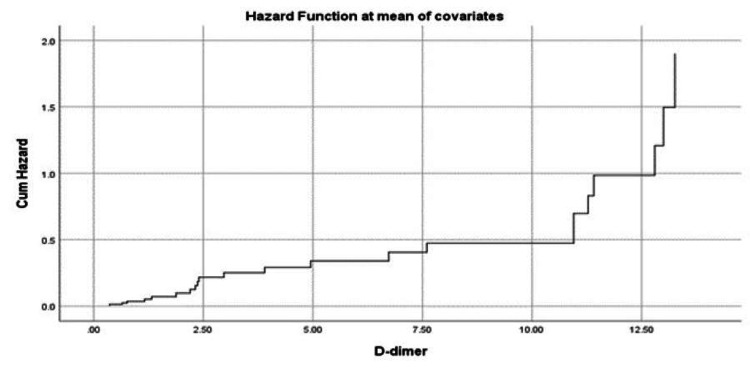
Mortality hazard among patients in association with ferritin concentration

**Figure 3:**
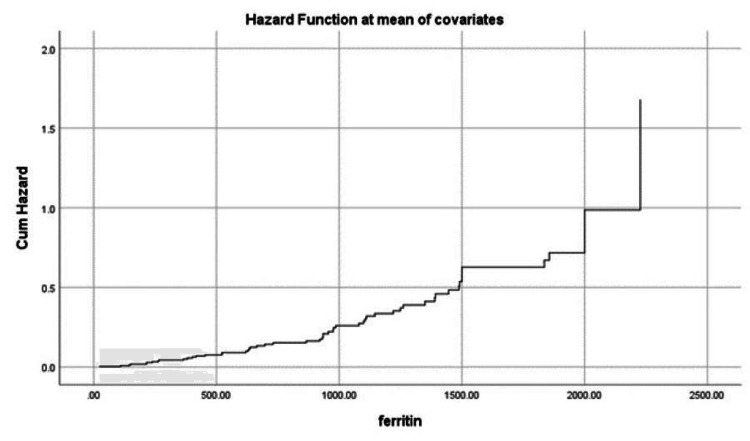
Mortality hazard among patients in association with age

The hazard of mortality increased significantly with increased D-dimer. An increase in D-dimer to more than 12.5 mcg/mL almost doubled the mortality risk (Figure 1). Additionally, ferritin could be an indicator of mortality risk, especially in those with ferritin concentrations of more than 2,000 ng/mL, where the risk was almost doubled (Figure 2). Advanced age was significantly associated with an increased risk of mortality, especially for those over the age of 80, with patients aged 100 having more than double the risk of mortality. The best survival outcomes were reported among patients younger than 60 years (Figure 3).

## Discussion

COVID-19 total mortality varies from 0.01% to 40%, depending on numerous risk factors such as age and comorbidities [13]. The vast majority of COVID-19 patients are asymptomatic or have moderate symptoms, but a small percentage may develop critical consequences such as acute respiratory distress syndrome, disseminated intravascular coagulation, multi-organ failure, sepsis, and finally death [14]. Numerous ideas have been proposed to explain the mechanisms behind the disparities in clinical outcomes, but this remains unanswered. Extended hospitalizations, an unpredictable course of the illness, and associated mortality fuel the hunt for biomarkers that may precisely, quickly, and effectively predict death in COVID-19 patients. This study analyzed retrospective data from 318 COVID-19 patients hospitalized in a tertiary care facility. Mortality increased significantly with age. In addition, the mean blood levels of ferritin and plasma D-dimer were considerably higher in COVID-19 non-survivors compared to survivors. The severity of the COVID-19 illness was likewise linked with the levels of these biomarkers.

Advanced age is an important independent predictor of death in sudden acute respiratory syndrome and Middle East respiratory syndrome [15,16]. Age was found to be a significant risk factor for mortality in this investigation of individuals with COVID-19. Earlier research in macaques showed that when injected with SARS-CoV-2, older macaques had stronger host innate responses, including a greater rise in the differential expression of genes involved in inflammation, compared to younger individuals, whereas type 1 interferon beta expression was suppressed [17]. Deficits in T-cell and B-cell activity as people age, as well as an increase in the production of type 2 cytokines, may lead to inadequate regulation of viral replication and more extended pro-inflammatory responses, all of which may adversely affect health outcomes [18].

Serum ferritin is a prognostic measure that predicts the progression of illness to more severe types [12,19]. A hyperinflammatory state with elevated ferritin develops in severe COVID-19. This is associated with higher death rates, multiple organ failures, and the necessity for admission to critical care units [12,20-23]. Chen et al. discovered that serum ferritin levels were considerably higher in individuals with severe COVID-19 compared to non-severe cases [24]. They discovered that a higher serum ferritin level was linked to a significant and life-threatening COVID-19 infection. This is similar to our result. Met et al. presented an iron chelator-based method to lower serum ferritin in COVID-19 [25]. According to them, reduced iron consumption may prevent COVID-19 aggravation, particularly in diabetics, because elevated ferritin is part of the normal course of diabetes. Lino et al. reported that serum ferritin at a cutoff of 1,873 ng/mL had a sensitivity of 68.4% and a specificity of 79.3% to predict death in COVID-19 [26]. This is comparable to our findings, which demonstrated that the mortality risk almost doubled at a concentration of more than 2,000 ng/mL. Ahmad et al. reported that serum ferritin at a cutoff of 574.5 ng/mL had a sensitivity of 80% and a specificity of 50% in predicting death in COVID-19 [27].

Some authors, however, have argued that ferritin is not a strong predictor of death, claiming that the value obtained while analyzing the area below the receiver operating characteristic curve demonstrated a poor capacity to discriminate mortality predictions. A similar result occurred when the negative and positive predictive values were examined [28]. According to Bolondi et al., ferritin is an early, nonspecific marker that increases in the early stages of the illness and falls after approximately a month. As a result, it might be beneficial in distinguishing individuals who may need hospital admission, but it is ineffective for patients who have already been admitted to the ICU [28-29]. In the case-control research of 100 patients by Zhou et al. [30], the COVID-19 cases had greater ferritin levels than the controls without COVID-19. In turn, these levels were greater among patients considered to have more serious conditions, as in the findings of this research. In the COVID-19 group, which was split into severe (seven men and five women) and moderate patients (22 men and 16 women), no significant variations in age or sex were seen [30]. In contrast, in another study that included a sample of 56 COVID-19-related deaths and 245 hospitalized patients, significant differences in age and duration of stay were seen between the two groups [31]. In that study, significant differences were found in terms of age, and a correlation was shown between age and increased mortality. It demonstrated a clear connection between higher blood ferritin levels and non-survivors [31]. In our study, a similar relationship was discovered.

One of the main findings of this study was that non-surviving patients had significantly higher mean levels of D-dimer (5.46) mcg/mL compared to surviving patients (1.55) mcg/mL. The gradual progression of severe sickness and the fatal result in COVID-19 patients is caused by coagulation dysfunction, which is defined by increased D-dimer and thrombi in the veins and arteries [32]. Excessive clotting and hypoxemia cause the high level of D-dimer in COVID-19. Moreover, COVID-19 patients with severe illnesses typically exhibit D-dimer elevation, which substantially correlates with death [9,33]. As a byproduct of fibrin breakdown, D-dimer can be used to predict deep vein thrombosis and pulmonary embolism [34].

The most recent research has shown that D-dimer is a useful prognostic sign for COVID-19 since infected individuals have higher levels than healthy patients; the higher the level, the worse the prognosis and course [35,36,37]. Additionally, individuals who are diagnosed with infection or sepsis in the emergency room have a documented link between high D-dimer levels and 28-day death [38]. A retrospective study by Fei et al. that included 191 patients with COVID-19 also revealed that a D-dimer level greater than 1 mcg/mL upon admission was associated with an 18-fold increased mortality risk [9]. Similar findings were made by Ning et al. concerning aberrant coagulation outcomes, particularly a noticeably raised D-dimer in fatalities due to COVID-19 [39]. In their analysis of 1,099 patients with laboratory-confirmed COVID-19 from more than 550 hospitals in China, Guan et al. observed that the D-dimer levels of non-survivors were substantially higher (2.12 mcg/mL on average) than those of survivors (0.61 mcg/mL on average) [38].

D-dimer elevation at the time of initial evaluation has been reported as one of the most common laboratory findings to predict coagulation-related problems. It has been identified in COVID-19 patients who needed to be hospitalized [35]. Huang et al. found that patients requiring critical care assistance had greater D-dimer levels upon admission than those who did not (median 0.5 mcg/mL) [40]. A multicenter retrospective study examined the incidence and severity of thrombotic complications in 400 hospitalized COVID-19 patients (144 in the ICU). That investigation discovered that individuals with higher D-dimer levels had severe symptoms and needed hospitalization [36,41].

In patients with COVID-19, an elevated D-dimer suggests a hypercoagulable condition, which might be caused by numerous different factors. First, a pro-inflammatory response is often severe, and the anti-inflammatory response is seldom under control during viral infections [42]. An increase in thrombin production due to endothelial cell dysfunction may be induced [43]. Second, the extreme hypoxia of COVID-19 can promote thrombosis by activating a hypoxia-inducible transcription factor-dependent signaling cascade and raising blood viscosity [44,45]. Third, risk factors for hypercoagulation or thrombosis have been defined as older age, underlying illnesses, long-term bed rest, and invasive therapy among hospitalized patients, especially patients with severe COVID-19 [46,47]. As proof, blockage and microthrombosis development in the pulmonary small arteries have been described in the lung organ dissection of critically ill patients with COVID-19 [48,49]. The fourth risk is sepsis-induced coagulopathy or even disseminated intravascular coagulation, which can occur in certain individuals [50]. An elevated D-dimer level has traditionally been linked to adverse outcomes [51,52]. Furthermore, D-dimer is considered a sensitive marker of thrombosis and hypercoagulability, and its elevation is also observed in patients with infection, surgery or trauma, malignancy, and other non-thrombotic diseases [53]. Its limited specificity is a disadvantage in some contexts, but it has been turned into a benefit in the evaluation of prognosis.

Limitations

This study has certain limitations. Firstly, this was a retrospective Ministry of Health Center study, which may limit the generalizability of the findings. Recording patient data only at the time of hospital admission could be another limitation. Additionally, because it was a retrospective observational study, not all laboratory tests were performed for all of the included patients.

## Conclusions

Advanced age, ICU admission, and the need for mechanical ventilation were associated with a higher risk of mortality in COVID-19 patients. D-dimer and ferritin concentration were found to be potential indicators of mortality risk, with higher levels of these markers significantly associated with increased mortality risk. In addition, our results suggest that older patients, particularly those over the age of 80, had a significantly higher risk of mortality. Patients younger than 60 had the best survival outcomes. These findings highlight the importance of early identification and targeted interventions for COVID-19 patients at high risk of mortality.
